# Cloning and purification of the first termicin-like peptide from the cockroach *Eupolyphaga sinensis*

**DOI:** 10.1186/s40409-016-0058-7

**Published:** 2016-01-28

**Authors:** Zichao Liu, Kehua Yuan, Ruopeng Zhang, Xuchen Ren, Xiaolong Liu, Shuhua Zhao, Dingkang Wang

**Affiliations:** Key Laboratory of Special Biological Resource Development and Utilization of Universities in Yunnan Province, Key Lab of Aquatic Ecological Restoration of Dianchi Lake in Kunming, Department of Biological Science and Technology, Kunming University, Kunming, 650214 China; Department of Oncology, Yan’an Hospital of Kunming City; Yunnan, Cardiovascular Hospital; and Yan’an Hospital of Kunming Medical University, Kunming, 650051 China; Department of Obstetrics and Gynecology, Shenzhen Maternal and Child Health Care Hospital, Affiliated to Southern Medical University, Shenzhen, 518028 China; Yunnan Key Laboratory of Fertility Regulation and Minority Eugenics, Yunnan Population and Family Planning Research Institute, Kunming, 650021 China; Kunming University, Puxin Road 2#, Kunming, Yunnan 650214 China; First Affiliated Hospital of Kunming Medical University, Xichang Road 295#, Kunming, Yunnan 650032 China

**Keywords:** *Eupolyphaga sinensis*, Cockroach, Termicin-like peptide, Es-termicin, Antifungal peptide

## Abstract

**Background:**

Termicin is an antimicrobial peptide with six cysteines forming three disulfide bridges that was firstly isolated from the salivary glands and hemocytes of the termite *Pseudacanthotermes spiniger*. In contrast to many broad-spectrum antimicrobial peptides, termicin is most active against filamentous fungi. Although more than one hundred complementary DNAs (cDNAs) encoding termicin-like peptides have been reported to date, all these termicin-like peptides were obtained from Isoptera insects.

**Methods:**

The cDNA was cloned by combination of cDNA library construction kit and DNA sequencing. The polypeptide was purified by gel filtration and reversed-phase high performance liquid chromatography (RP-HPLC). Its amino acid sequence was determined by Edman degradation and mass spectrometry. Antimicrobial activity was tested against several bacterial and fungal strains. The minimum inhibitory concentration (MIC) was determined by microdilution tests.

**Results:**

A novel termicin-like peptide with primary structure ACDFQQCWVTCQRQYSINFISARCNGDSCVCTFRT was purified from extracts of the cockroach *Eupolyphaga sinensis* (Insecta: Blattodea). The cDNA encoding Es-termicin was cloned by cDNA library screening. This cDNA encoded a 60 amino acid precursor which included a 25 amino acid signal peptide. Amino acid sequence deduced from the cDNA matched well with the result of protein Edman degradation. Susceptibility test indicated that Es-termicin showed strong ability to kill fungi with a MIC of 25 μg/mL against *Candida albicans* ATCC 90028. It only showed limited potency to affect the growth of Gram-positive bacteria with a MIC of 200 μg/mL against *Enterococcus faecalis* ATCC 29212. It was inactive against gram-negative bacteria at the highest concentration tested (400 μg/mL). Es-termicin showed high sequence similarity with termicins from many species of termites (Insecta: Isoptera).

**Conclusions:**

This is the first report of a termicin-like peptide isolated from *E. sinensis* that belongs to the insect order Blattodea. Our results demonstrate the diversity of termicin-like peptides, as well as antimicrobial peptides in insects.

## Background

Host innate immune system is critical for insects against pathogenic microbes, one of its major components are the antimicrobial peptides (AMPs) [1]. They are produced by the immune deficiency signaling pathway, in response to the bacterial cell wall peptidoglycan fragments [2]. Since the first insect AMP was purified from the pupae of moth *Hyalophora cecropia* in 1980, more than 150 insect AMPs have been identified [3, 4]. Many of these peptides showed broad-spectrum antimicrobial activity against gram-positive bacteria, gram-negative bacteria and fungi [5–7].

Termicins are a family of AMPs with strong antifungal activities. The first known termicin was isolated from the termite *Pseudacanthotermes spiniger* [8]. It is highly expressed in the hemocyte granules and salivary glands of termites. More than 100 cDNAs encoding termicin-like peptides from different termite species have been reported to date. However, no termicin-like peptide has been described from other insect species.

*Eupolyphaga sinensis* is an insect widely distributed throughout China and, as most cockroaches, it inhabits leaf litter, dead wood and humus-rich soil nearby corners of human buildings. They are nocturnal insects that look for rice bran, corn flour and other nutritious debris for food during the night. Female cockroaches are wingless with oblate shell on the back. This species has been used in Chinese traditional medicine for many years. According to folk medical practices, it is thought to enhance immune response and improve blood circulation. In recent years, researchers have discovered cancer cell inhibition, immunoregulatory and angiogenic effects from extracts of *E. sinensis* [9–13]. Moreover, a few bioactive molecules – including adhesion inhibitors, fibrinogenolytic and plasminogen-activating proteins – have been reported [14–16]. Nevertheless, studies focused on the innate immune system of this species are scarce. In this study, we present the isolation, characterization and cloning of the first cockroach AMP.

## Methods

### Sample preparation

Adult insect specimens of *E. sinensis* of both genders (*n* = 2,800) were purchased from Shandong Province of China. Isolates extracted from the whole bodies of *E. sinensis* except the chitin shells and alimentary canals were used for peptide purification. These isolates were homogenized in Tris–HCl buffer (20 mM Tris–HCl, pH 7.6, containing 0.1 M NaCl and 5 mM EDTA-2Na). After centrifugation, the homogenate supernatant was lyophilized and stored at −80 °C until used to purify active components. Animal care and handling were conducted in accordance with the requirements of the Ethics Committee of Kunming University.

### Peptide purification

Lyophilized sample of *E. sinensis* was dissolved in 3 mL of Tris–HCl buffer, and then loaded on a Sephadex G-50 (Superfine, GE Healthcare) gel filtration column which had been equilibrated with the same buffer at a flow rate of 9 mL/h. Fractions were collected every 20 min. The absorbance of each tube was monitored at 280 nm. Fractions containing antimicrobial activity were pooled and applied to a C18 reverse-phase high performance liquid chromatography column (RP-HPLC, Hypersil BDS C18, 4.0 × 250 mm, Elite, China) which was equilibrated with 0.1 % (v/v) trifluoroacetic acid/water and gradient eluted with 0.1 % (v/v) trifluoroacetic acid/acetonitrile at a flow rate of 1 mL/min. The absorbance of isolated fractions was monitored at 215 nm.

### Peptide structural analysis

Mass spectrum and N-terminal sequencing of purified peptide were carried out according to our previous reported methods [17]. The observed molecular mass and the purity of sample were determined on an Autoflex Speed TOF/TOF mass spectrometer (Bruker Daltonik GmbH) in linear mode. All procedures were carried out according to manufacturer’s standard protocols and the data were analyzed by the software package provided by the manufacturer. The peptide sequence was determined by Edman degradation on a PPSQ-31A protein sequencer (Shimadzu, Japan) according to the standard glass fiber disk (GFD) method instructed by manufacturer’s protocol. Both mass spectrum and N-terminal sequence determination were carried out in Kunming Institute of Zoology, the Chinese Academy of Sciences.

### cDNA synthesis and cloning

Total RNA was extracted from the whole bodies except the chitin shell and alimentary canals, corresponding to the above isolation. cDNA library was constructed mainly according to our previously reported methods [17]. Briefly, the total RNA was extracted with TRIzol Reagent (Invitrogen, USA). Isolation of mRNA from the total RNA was performed with Oligotex mRNA mini kit according to the standard protocol (Qiagen, USA), and 0.1 μg mRNA was used for library construction. The synthesis of cDNA was performed using a SMART PCR cDNA synthesis kit (Clontech, USA). The first strand of cDNA was synthesized using the SMARTScribe MMLV Reverse Transcription system with the primer pair CDS III/3’ PCR primer, 5’-ATTCTAGAGGCCGAGGCGGCCGACATG-D (T)30 N-1 N-3’ (N = A, G, C, or T; N-1 = A, G, or C) and SMART IV Oligonucleotide, 5’-AAGCAGTGGTATCAACGCAGAGTGGCCATTACGGCCGGG-3’. The second strand was produced using advantage polymerase and primer pair CDS III/3’ PCR primer and 5’ PCR primer 5’-AAGCAGTGGTATCAACGCAGAGT-3. The PCR was performed as follows: 2 min at 94 °C followed by 30 cycles of 30 s at 92 °C, 30 s at 55 °C, and 60 s at 72 °C, and then a final extension step of 72 °C for 10 min. The PCR products were ligated into pMD^TM^ 19-T vector [TaKaRa Biotechnology (Dalian) Co., Ltd., China] and then transformed into *E. coli* DH5α competent cells. A cockroach cDNA library was constructed. Clones with the cDNA insert > 300 bp were randomly chosen to carry out DNA sequencing on an Applied Biosystems DNA sequencer (ABI 3730XL, USA).

### Sequence, structure, and evolutionary analyses

The signal peptide was predicted with the SignalP 4.1 program (http://www.cbs.dtu.dk/services/SignalP/). The signal peptide cleavage site was confirmed by the determined sequence of mature Es-termicin. The amino acid sequence of mature polypeptide was compared against public databases (http://blast.ncbi.nlm.nih.gov/Blast.cgi). The resulting sequences were aligned using the ClustalW2 (http://www.ebi.ac.uk/Tools/clustalw2/index.html). The alignment was imported into MEGA software to construct phylogenetic tree by the maximum likelihood method [18].

### Antimicrobial assays

Several bacterial and fungal strains for antimicrobial assays including gram-positive bacterium *Staphylococcus aureus* (ATCC25923), *Enterococcus faecalis* (ATCC29212), gram-negative bacterium *Haemophilus influenza* (ATCC49767), *Pseudomonas aeruginosa* (CMCCB1010) and fungus *Candida albicans* (ATCC2002, 90028, 90030), *Candida parapsilosis* (ATCC 22019) and four clinical isolates [*S. aureus* (IS 10#), *S. aureus* (IS 39#), *Escherichia coli* (IS 121#) and *Pseudomonas aeruginosa* (IS 320#)] were kindly provided from Kunming Medical College. Microorganisms were first grown in Luria-Bertani (LB) broth to an absorbance of 0.5 at 600 nm. A 10 μL aliquot of LB containing microorganisms was then mixed with 8 mL of fresh LB broth with 0.7 % agar and poured over a 90 mm Petri dish containing 25 mL of 1.5 % agar in LB broth. Antimicrobial assay was carried out according to a microdilution method as previously described [19] by dropping a 20 μL aliquot of the tested sample with different concentration in 0.9 % NaCl onto the surface of the top agar. After overnight incubation at 37 °C, the antimicrobial activity was determined according to the growth-inhibitory zone on the surface of the top agar. Antimicrobial ability was evaluated by determining minimal inhibitory concentration (MIC) in liquid LB medium. At MIC, no visible growth was observed.

### Hemolytic assays

Human red blood cells were provided by Kunming Blood Center of Yunnan Province. Red blood cells in Alsever’s solution (in g/L: NaCl, 4.2; trisodium citrate dihydrate, 8.0; citric acid monohydrate, 0.55; D-glucose, 20.5) were used to test hemolytic ability of Es-termicin according to the methods reported by Bignami [20]. Es-termicin was serially diluted and incubated with red blood cells at 37 °C for 30 min. After centrifugation, the absorbance in the supernatant was measured at 540 nm. Maximum hemolysis was determined by adding 1 % Triton X-100 to the cell samples.

## Results

### Purification of termicin from cockroach

**Fig. 1:**
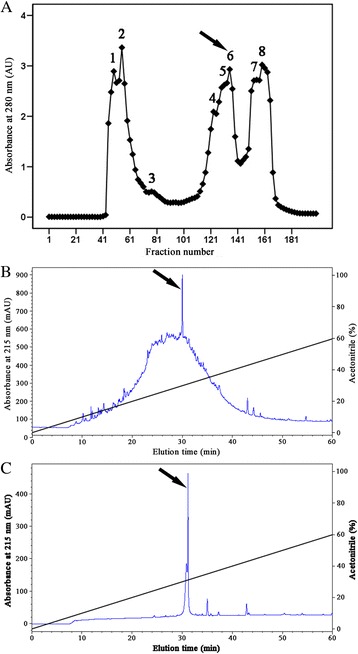
Purification of Es-termicin from cockroach *E. sinensis*. **a** Gel filtration chromatography of *E. sinensis* sample on a Sephadex G-50 (superfine) column. The column (2.6 × 100 cm) was pre-equilibrated with Tris–HCl buffer (20 mM Tris–HCl, pH 7.6, containing 0.1 M NaCl) at a flow rate of 9 mL/h. Fractions were collected every 20 min. The absorbance of each tube was monitored at 280 nm. **b** Fraction 6 from (**b**) was pooled and applied on a C18 reverse-phase high performance liquid chromatography column (RP-HPLC, Hypersil BDS C18, 4.0 × 250 mm, Elite, China) equilibrated with 0.1 % (v/v) trifluoroacetic acid/water. The elution was performed with the indicated gradient of 0.1 % (v/v) trifluoroacetic acid/acetonitrile at a flow rate of 1 mL/min. **c** The fraction indicated by an arrow from (**b**) was further purified by the same RP-HPLC C18 column again. Fractions with antimicrobial activity are indicated by an arrow

The pooled cockroach extracts were separated into eight fractions (named P1 through P8) by gel filtration on a Sephadex G-50 column (Fig. 1a). The antimicrobial activity was mainly concentrated in P6 that was collected and applied to a RP-HPLC C18 column (Fig. 1b). The interest fraction indicated by an arrow in Fig. 1b was further purified by the same RP-HPLC C18 column again. The fraction indicated by an arrow in Fig. 1c displayed antimicrobial activity.

### Amino acid sequencing and structure characterization

**Fig. 2:**
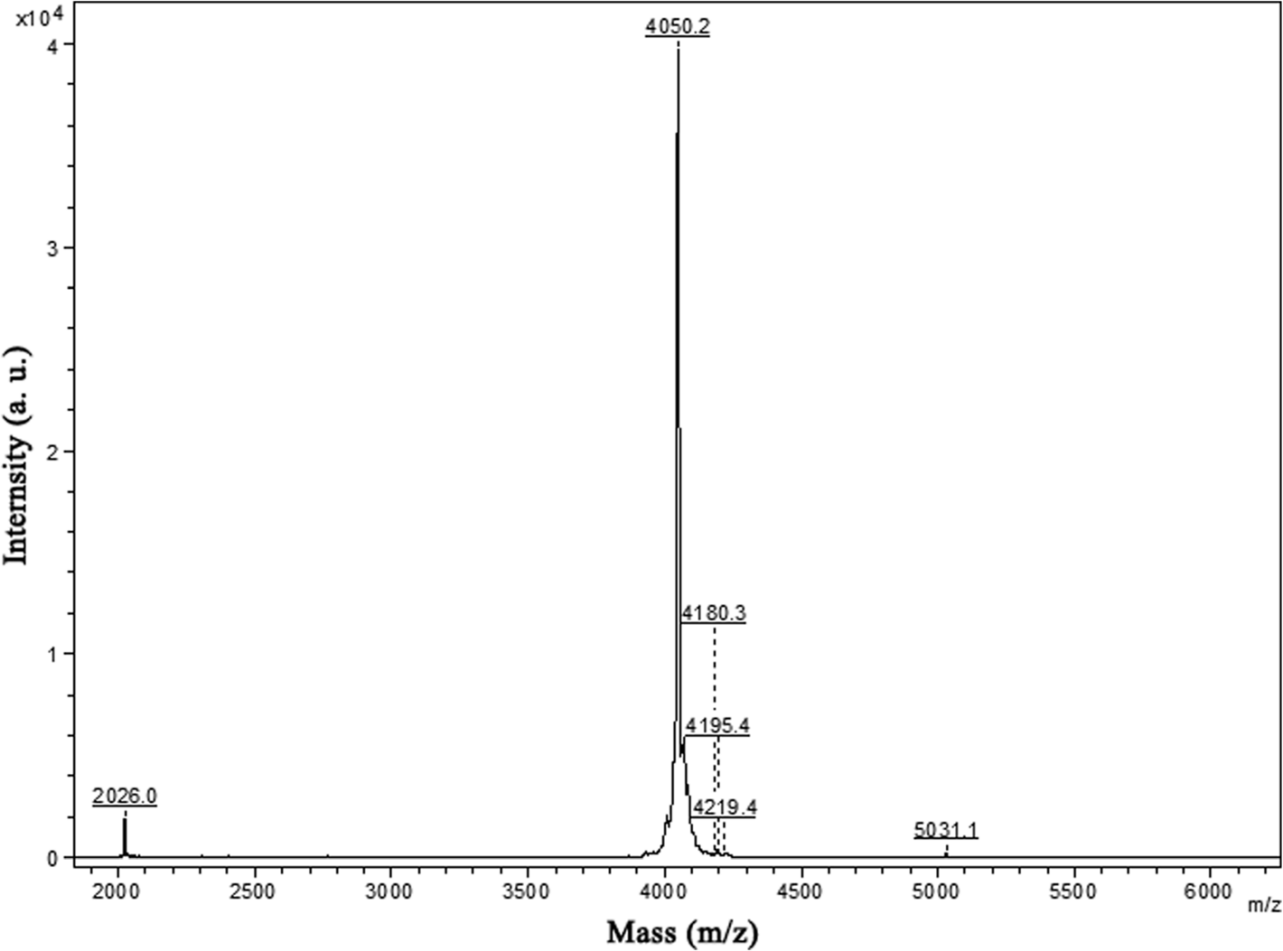
Autoflex Speed TOF mass spectrum of Es-termicin

**Fig. 3:**
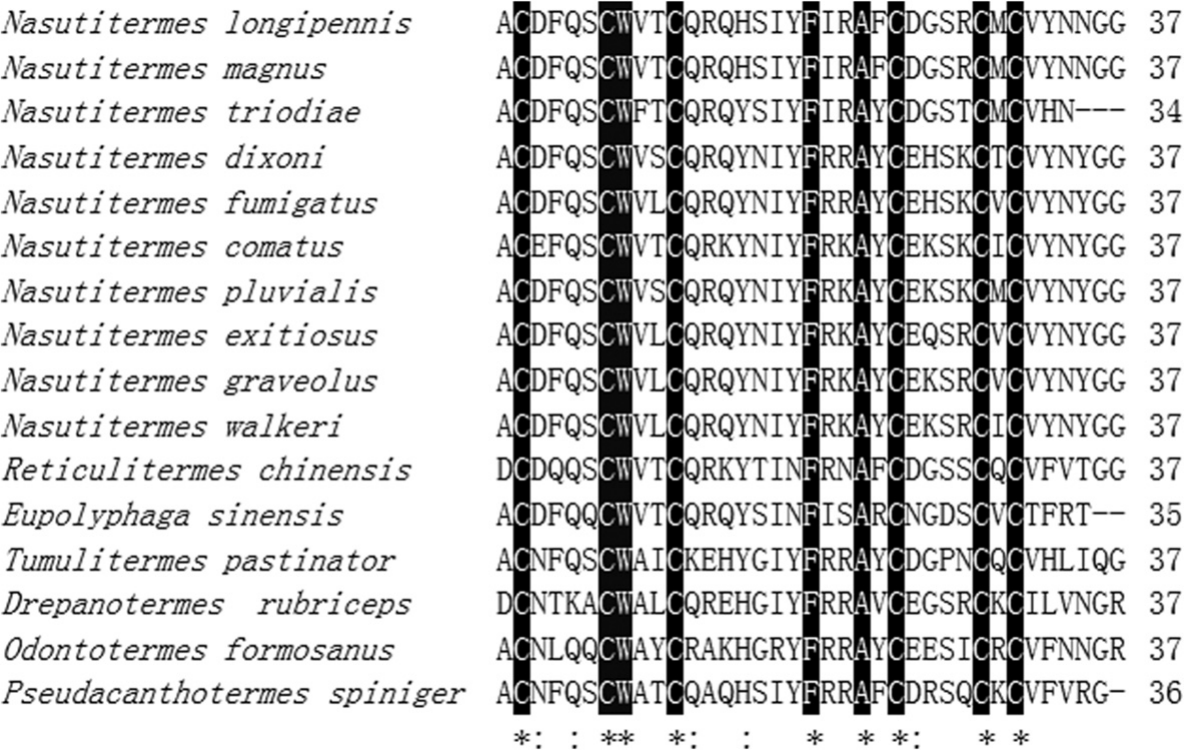
Sequence alignment of Termicins. The sequences used in the alignment are from *Eupolyphaga sinensis* (this work), *N. comatus* (GenBank accession no. AY642891), *N. dixoni* (GenBank accession no. AY642893), *N. exitiosus* (GenBank accession no. AY642894), *N. fumigatus* (GenBank accession no. AY642896), *N. graveolus* (GenBank accession no. AY642897), *N. longipennis* (GenBank accession no. AY642898), *N. magnus* (GenBank accession no. AY642899), *N. pluvialis* (GenBank accession no. AY642900), *N. triodiae* (GenBank accession no. AY642902), *N. walkeri* (GenBank accession no. AY642904), *Tumulitermes pastinator* (GenBank accession no. AY642908), *Drepanotermes rubriceps* (GenBank accession no. AY642909), *Pseudacanthotermes spiniger* (GenBank accession no. P82321), *Reticulitermes chinensis* (GenBank accession no. FJ184583), *Odontotermes formosanus* (GenBank accession no. FJ184507), *Reticulitermes virginicus* (GenBank accession no. GU906793), *Reticulitermes flavipes* (GenBank accession no. GU906820). Cysteinyl residues are indicated by asterisks. Amino acid residues are numbered on the right

**Fig. 4:**
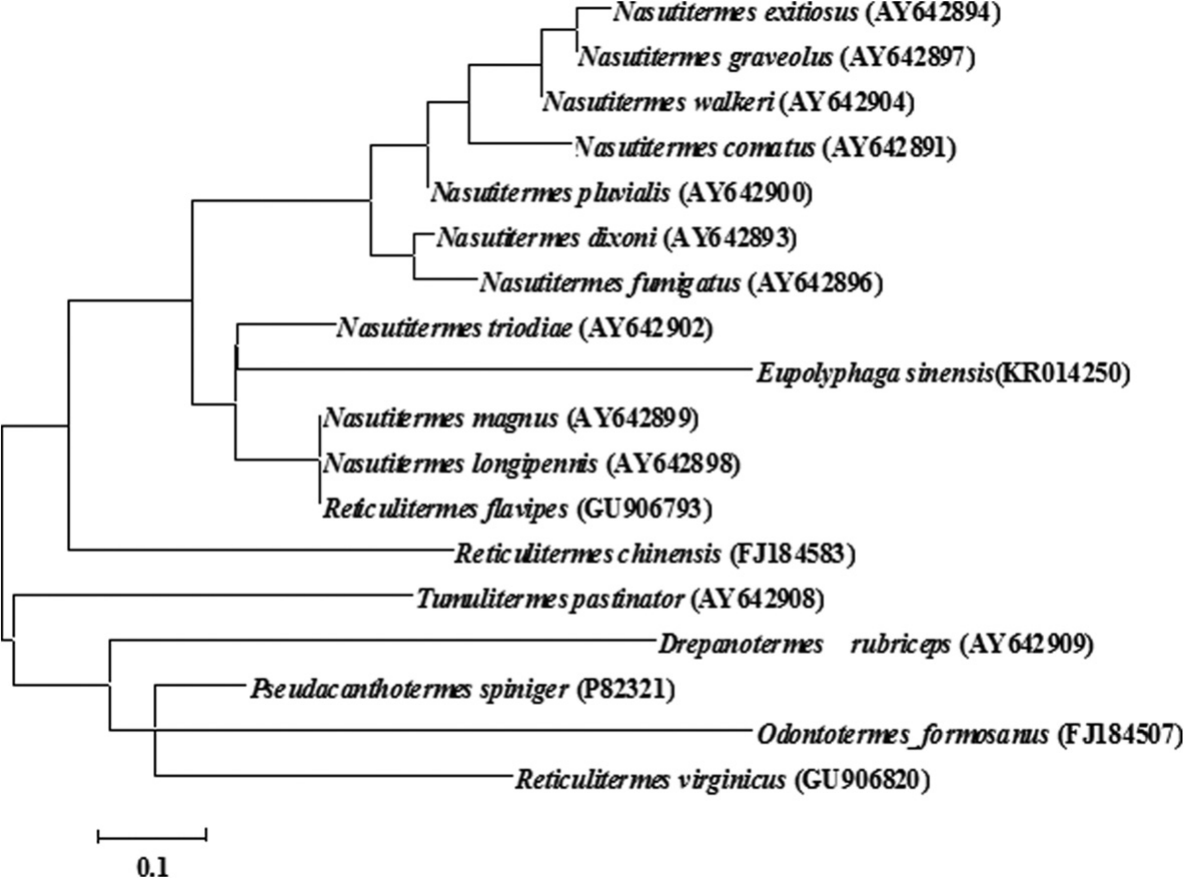
Phylogenetic tree of termicins. A distance tree (maximum-likelihood) for mature termicins was constructed by MEGA 6. The species name and GenBank accession number are indicated in the tree. Numbers at the nodes represent bootstrap proportions on 1000 replicates. Scale bars are in nucleotide substitutions per site

The fraction indicated by an arrow in Fig. 1c eluted from the RP-HPLC was seemly a purified peptide because it recorded a single peak at 4050.2 MH^+^ by mass measurement (Fig. 2). This fraction was pooled and subjected to amino acid sequence analysis by automated Edman degradation. The resulting sequence is ACDFQQCWVTCQRQYSINFISARCNGDSCVCTFRT which was named Es-termicin. The mature protein sequence of Es-termicin was compared with the sequences of other termicins using the ClustalW2 software. High degree of similarity was observed among these sequences. Mature Es-termicin shared 68.8 % identity with that of termicin-like peptide from *Nasutitermes triodiae* (Fig. 3). All these sequences have six conserved cysteinyl residues. To determine the relationship between Es-termicin and other termicins from termites, a phylogenetic tree was constructed based on comparison of amino acid sequences of mature termicins. Phylogenetic tree indicated that Es-termicin is most structurally closed to that of *N. triodiae* (Fig. 4).

### cDNA cloning

**Fig. 5:**
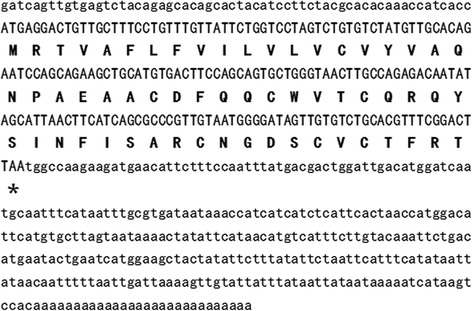
Amino acid sequences of Es-termicin and its cDNA sequence from cockroach *E. sinensis*. 5’and 3’ uncoding regions are shown in lowercase letters. The stop codon is indicated by asterisk. The signal peptide is shown with underlines. Mature protein sequence is boxed

The cDNA encoding Es-termicin was screened by cDNA library constructing and sequencing (GenBank accession: KR014250). This cDNA contained an open reading frame which encoded a polypeptide composed of 60 amino acids, including the mature Es-termicin and a 25 amino acid signal peptide (Fig. 5). This signal peptide was predicted with the SignalP 4.1 program (http://www.cbs.dtu.dk/services/SignalP/) and confirmed by the sequence Edman degradation. The amino acid sequences deduced from the cDNA sequences match well with the amino acid sequences determined by Edman degradation.

### Bioactivities of the Es-termicin

Isolated Es-termicin was tested on various filamentous fungi, and gram-positive and gram-negative bacteria. Es-termicin had marked activity against *Candida albicans*, but was less active against *Candida parapsilosis* (Table 1). It was inactive against the gram-positive and gram-negative bacteria at the highest concentration tested (400 μg/mL), besides weak activity against *Enterococcus faecalis*. Termicin had a similar level of activity against three yeast strains tested (*Candida albicans*). No activity against gram-negative bacteria was detected even at 400 μg/mL.

**Table 1:** The antimicrobial activity of Es-termicin

Microorganisms	^a^MIC (μg/mL)	
	Ampicillin	Termicin
Gram-positive bacteria		
*Staphylococcus aureus* ATCC 25923	4	ND
*Enterococcus faecalis* ATCC 29212	32	200
*Staphylococcus aureus* (IS 10#)	ND	ND
*Staphylococcus aureus* (IS 39#)	ND	ND
Gram-negative bacteria		
*Haemophilus influenza* ATCC 49767	32	ND
*Pseudomonas aeruginosa* CMCCB1010	32	ND
*Escherichia coli* (IS 121#)	ND	ND
*Pseudomonas aeruginosa* (IS 320#)	ND	ND
Fungi		
*Candida albicans* ATCC 2002	32	50
*Candida albicans* ATCC 90028	32	25
*Candida albicans* ATCC 90030	32	50
*Candida parapsilosis* ATCC 22019	32	100

Ampicillin was used as a positive control. ND means no activity was detectable under the tested dosage of samples up to 400 μg/mL. ^a^MIC, minimum inhibitory concentration required for total inhibition of cell growth in liquid medium

Human red blood cells were used to check for hemolytic capability in our experiments. Es-termicin had little hemolytic activity on red blood cells even with peptide concentration up to 400 μg/mL.

## Discussion

Although more than one hundred mRNAs encoding termicin-like peptides have been reported from more than ten species of termites, only the protein of termicin from *Pseudacanthotermes spiniger* was purified [8, 21–23]. In the current study, a novel termicin-like AMP called Es-termicin was isolated and characterized from cockroach *E. sinensis*. Its primary structure ACDFQQCWVTCQRQYSINFISARCNGDSCVCTFRT was determined by Edman degradation and mass spectrometry. This sequence showed high similarity with that of termicin from deduced protein sequences of mRNA from these species of termites.

Termicin is firstly isolated from termite *Pseudacanthotermes spiniger*. In contrast to many AMPs that have rather broad spectrum of inhibiting activity, termicin mainly inhibits filamentous fungi and yeast. It only has limited potency to affect the growth of gram-positive bacteria.

Biological function depends on the structure of the polypeptide. The antifungal activity of termicin is also determined by its special structure. Termicin contains six cysteines forming three disulfide bridges with cysteine pairing pattern of Cys(1)-Cys(4), Cys(2)-Cys(5), Cys(3)-Cys(6) [8]. In this study, the difference (5.38 Da) between the observed molecular mass (4050.2 MH^+^) and the calculated mass of the peptide (4055.58 MH^+^) by the ExPASy MW/pI tool (http://www.expasy.ch/tools/pi_tool.html) suggests that Es-termicin also forms three disulfide bridges. The tertiary structure of termicin contains an α-helical segment (Phe4-Gln14) and a two-stranded (Phe19-Asp25 and Gln28-Phe33) antiparallel β-sheet forming a cysteine-stabilized αβ motif which is similar to that of insect defensins [24, 25]. Defensins are selectively active against gram-positive bacteria, first reported from immunized larvae of the dipteran insect *Phormia terranovae* [26]. However, with the exception of cysteine pairing pattern, termicin shows little sequence homology with insect defensins. This structural comparison suggests that cysteine pairing pattern alone does not explain the difference between antifungal and antibacterial activity.

Insects have enough chemical defense systems including AMPs to survive in the continuously varying environment with a variety of pathogenic microorganisms [27, 28]. Many insect AMPs, such as defensins, cecropins, proline-rich peptides, and attacins, have been found in more than two insect orders [3]. However, termicins have been identified only in Isoptera before. This report presents the first termicin-like peptide from Blattodea insects. The result demonstrates the diversity of termicin-like peptides, as well as antimicrobial peptides in insects. AMPs are being considered as new drug candidates in the post-antibiotic era [29]. More diversity of AMPs provides more opportunities for designing effective antimicrobial agents.

## Conclusion

In this study, a novel peptide named Es-termicin with an amino acid sequence ACDFQQCWVTCQRQYSINFISARCNGDSCVCTFRT was identified from cockroach *E. sinensis*. It showed strong activity against fungi and weak activity against some bacteria. Termicins were reported only from Isoptera insects before. This is the first report of termicin-like peptide from *E. sinensis* which belongs to the insect order Blattodea. Our results demonstrate the diversity of termicin-like peptides, as well as antimicrobial peptides in insects.

### Ethics approval

Animal care and handling were conducted in accordance with the requirements of the Ethics Committee of Kunming University, China.
